# Reframing Aging in the SDGs Era: The Role of Nonprofits in Helping Older Persons and Holding Governments to Account

**DOI:** 10.1093/geroni/igaa057.2986

**Published:** 2020-12-16

**Authors:** Mika Maromoto

**Affiliations:** HelpAge International, Washington, District of Columbia, United States

## Abstract

With five years gone since the adoption of the UN Agenda for Sustainable Development, the global community is moving far too slowly toward achieving its 2030 objectives for older persons. Achieving proper rights and wellbeing for older persons requires strategic and concerted action. The WHO Decade of Healthy Ageing initiative is one useful step toward defining the diverse physical, psychological, social and healthcare needs of older persons. But government leadership remains weak, and the nonprofit sector plays the key role spearheading advocacy efforts toward the SDG motto of “leave no one behind.” The urgent needs of older persons – whether fighting ageism, upholding the rights of people living with dementia, or promoting lifelong learning and contributions to society - can best be met by societies that “reframe aging” and craft creative and innovative solutions. This presentation showcases NGOs that are leading the way in cultivating such transformative solutions.

